# Implications of living evidence syntheses in health policy

**DOI:** 10.2471/BLT.23.290540

**Published:** 2024-09-02

**Authors:** Samantha Chakraborty, Tanja Kuchenmüller, John Lavis, Fadi El-Jardali, Laurenz Mahlanza-Langer, Sally Green, Ludovic Reveiz, Victoria Carter, Emma McFarlane, Cheryl Pace, Lisa Askie, Fiona Glen, Tari Turner

**Affiliations:** a Cochrane Australia, School of Public Health and Preventive Medicine, Monash University, 553 St Kilda Road, Melbourne 3004, Victoria, Australia.; b Science Division, World Health Organization, Geneva, Switzerland.; c McMaster Health Forum, Health Research Methods, Evidence, and Impact, McMaster University, Toronto, Canada.; d Health Policy and Management Department, American University of Beirut, Beirut, Lebanon.; e Pan-African Collective for Evidence, Johannesburg, South Africa.; f Science and Knowledge Unit, Evidence and Intelligence for Action in Health Department, Pan American Health Organization, Washington, DC, United States of America.; g National Institute for Health and Care Excellence, Manchester, England.; h Healthcare Improvement Scotland, Glasgow, Scotland.

Living evidence syntheses are systematically appraised and continually updated summaries of research evidence,^1^ such as living evidence-based guidelines,^2^ living systematic reviews,^3^ living evidence maps and living policy briefs.^4^ These syntheses are driven by the commitment to ongoing, frequent surveillance for new research evidence, where frequency is decided by the likelihood of new evidence emerging, anticipated changes in policy or practice contexts change, or the need and urgency for decision-making.^1^^,^^3^ For example, at the height of the coronavirus disease 2019 (COVID-19) pandemic, living clinical guidelines for COVID-19 treatment were updated weekly to rapidly incorporate emerging evidence to inform practice and policy. As these living syntheses are revised, when new evidence emerges or the context evolves, they differ from updated evidence syntheses, such as the *World Health Organization* *Model List of Essential Medicines*, which are updated on predetermined timelines, annually, bi-annually or less frequently. 

In the last 3–5 years, the use of living evidence syntheses has increased, with living evidence-based guidelines on maternal and child health,^5^ diabetes^6^ and COVID-19,^2^^,^^7^ as well as hundreds of living systematic reviews now published. Methods for producing robust living evidence syntheses have progressed considerably;^8^^,^^9^ however, several aspects of the approach are underexplored. These aspects include when to use a living evidence approach, how to implement findings from these syntheses into policy and practice, and their suitability for public health and policy questions. In this paper, we draw on our experiences as producers and users of living evidence syntheses for policy in diverse global and local settings to articulate the potential ways in which these syntheses could strengthen evidence-based health policy-making, some of the challenges in their use, and next steps towards realizing their potential for health policy-making.

## Producers, intermediaries and users

Global and national organizations, such as the World Health Organization (WHO) and Cochrane, as well as individual researchers, produce and maintain living evidence syntheses to serve as global public goods. These goods include living systematic reviews, standards or other guidance that respond to new evidence. Local users – such as individuals within governments and health organizations – frequently with intermediaries, such as the McMaster Health Forum, Pan African Collective for Evidence or members of the WHO Evidence-informed Policy Network, use these living evidence syntheses to inform and shape health policy decisions. As such, producers are responsible for ensuring that living evidence syntheses are credible, accessible and timely. Intermediaries are responsible for supporting users to identify priority topics for living evidence syntheses, finding relevant syntheses or collectively adapting global syntheses. Users are responsible for applying evidence to address specific policy questions and strengthening domestic evidence infrastructure to enable the effective development and use of living evidence syntheses. All three groups are responsible for demonstrating the benefit of living evidence in health policy.

## Benefits

Evaluations, predominantly focused on COVID-19, have shown that living evidence syntheses support evidence-based care and boost clinician confidence, by providing rapid access to up-to-date, reliable evidence summaries.^10^ Similar benefits apply to health policy. Compared to less frequently or irregularly updated sources of evidence, living evidence syntheses provide timely access to the most up-to-date evidence, increasing policy-makers’ confidence in their own decisions.^11^ These syntheses can be particularly valuable when the evidence base is dynamic, emerging quickly, and the policy context is evolving, such as in cases of rising noncommunicable diseases, climate change impacts on health or pandemic responses.^10^^,^^11^

Importantly, collaborative and technological solutions that enable the production of living evidence syntheses (such as the Alliance for Living Evidence; Epistemonikos® and Covidence®) may also benefit health systems and potentially save resources by reducing duplication of effort to produce evidence syntheses. This benefit is likely because the underpinning rigour of living evidence syntheses, enabled by these initiatives, enhances the credibility of evidence syntheses outputs. The ongoing maintenance of these syntheses means that sources are up-to-date when knowledge users need them.^10^

One additional benefit of living evidence syntheses is the iterative, repeated nature of contact between producers and users. This contact provides opportunities to progressively refine and co-develop products and processes to ensure that these products continue to meet user needs. This continual engagement between users and producers may improve policy-making through greater awareness and understanding of different roles and perspectives, and as a by-product of the sustained relationships that successful living evidence syntheses require.

## Challenges 

Using living evidence syntheses to inform health policy across countries and across national and local settings for a range of health topics^2^^,^^4^^,^^7^ has also raised several new challenges. Foremost are determining when to produce these syntheses (compared with traditional evidence syntheses) in policy decisions, and how to translate their outputs into policy and practice. Unanswered questions include how to ensure acceptability of policy decisions that are based on living evidence, where the evidence base may evolve after a policy is made or, if adapting policy in response to emerging evidence, how to facilitate implementation of living policy into practice.

The COVID-19 pandemic prompted living evidence syntheses production across clinical care, public health and health systems.^12^ During the pandemic, their production was amplified to supply responsive, continual updates to decision-makers who needed to act urgently, with significant uncertainty, within rapidly evolving health, economic, social and research landscapes. Living evidence syntheses informed health policy by guiding decisions on the purchase of drugs to treat COVID-19,^10^ mask-wearing,^7^ travel requirements^7^ and more.^7^^,^^10^ Living evidence syntheses enabled users, intermediaries and producers to quickly identify and respond to research gaps, and empowered local users to set policy agendas and priorities that were underpinned by reliable, relevant evidence that was responsive to the changing context.^7^ While the urgency and necessity of the pandemic motivated local decision-makers to commission living evidence syntheses for policy questions, the criteria for deciding whether or not to use these methods is unclear. Such decisions may be influenced by priority of the topic for decision-making, uncertainty in the existing evidence, expectation of emerging evidence, changing policy context and the presence of an enabling environment for living evidence syntheses-informed policy uptake.^1^

Furthermore, outside the context of a pandemic, during which local users are willing to regularly engage with living evidence syntheses, evidence on effective translation of such syntheses into policy is lacking, particularly across users with diverse language, context and evidence needs. For example, it is unclear whether knowledge translation strategies (such as dissemination and consultation) for static evidence syntheses are applicable in a living context, and if not, which strategies are more appropriate for translation of living evidence syntheses into policy and practice.

Additional challenges stem from inconsistent updates of these syntheses, misaligned expectations and a lack of consensus about optimal frequency of surveillance for new research. During the COVID-19 pandemic, a recurrent challenge for intermediaries was the reliance on global producers to ensure that evidence syntheses were rigorously produced and maintained in a truly living, up-to-date state. Local users wanted and expected prompt access to these syntheses,^10^ and looked to intermediaries to provide and maintain contextualized syntheses within specific timelines. These expectations contrasted with typical academic review and publication timelines – for example with peer-reviewed journals – and exposed challenges for academic and publication mechanisms to enable timely dissemination of updates that were generated from living evidence syntheses. When global producers failed to continually produce these syntheses, it eroded trust between intermediaries and local users, as intermediaries were unable to meet their commitments to local users.

Finally, during the COVID-19 pandemic intermediaries needed to be highly adaptable, adjusting the pace of surveillance, topics and questions for these syntheses, reporting methods and dissemination activities and products as contexts, issues and evidence evolved. Doing so meant absorbing uncertainty in the quantity and timing of evidence screening and retrieval, and subsequent evidence appraisal and syntheses. To avoid the risk of not having reliable up-to-date evidence to inform policy, many intermediaries, including the National Clinical Evidence Taskforce and the Pan American Health Organization, adopted dual roles (as living evidence producer and intermediary), developing their own living evidence syntheses, sharing these internationally and assisting other intermediaries to contextualize them for local policy. This role required funding and capacity to both produce living evidence syntheses and contextualize these syntheses for local use, in addition to collaborating with evidence producers and users from other contexts. During the COVID-19 pandemic, we had both funding and methodological capacity to produce and enable wide translation of living evidence syntheses. However, the significant resources required raises equity issues for access to these syntheses in resource-constrained contexts, such as low- and middle-income countries.

## Conclusion

Living evidence syntheses have the potential to support health policy by providing reliable, up-to-date evidence that rapidly addresses high-priority policy areas where the evidence base is quickly changing over time. However, there is still much to learn about improving this approach to maximize the societal benefits. In addressing this need, consensus at a global level will be valuable for defining living evidence syntheses, and to determine what safeguards and other structures are necessary to ensure that these syntheses are conducted rigorously and ethically, and are accessible to populations worldwide. We suggest six broad areas of work to support and enhance the widespread use of living evidence syntheses in health policy. First, developing criteria for when to use living evidence syntheses approaches to inform policy and optimal frequencies of updates. Second, understanding what topics and types of research are suited to a living approach. Third, understanding the implications for users of living evidence syntheses and how to implement policy informed by these syntheses. Fourth, understanding how these syntheses affect English-language and high-income country biases. Fifth, developing online platforms that enable equitable access to curated high-quality syntheses and study level data so that these can be accessed by users globally. Finally, developing, evaluating and implementing technological solutions, including artificial intelligence, to produce living evidence syntheses ethically, efficiently and accurately.

Challenges to developing and using these methods for policy will be overcome through funding and collaboration. We invite living evidence producers, intermediaries and users, who are also committed to making living evidence approaches useful in health policy, to work collaboratively to improve the living evidence syntheses methods to enable evidence-informed health policy.
